# Complete genome sequencing of the fowlpox virus vaccine strain from the Livestock Research Institute, Bangladesh

**DOI:** 10.1128/mra.00502-26

**Published:** 2026-06-15

**Authors:** Mst. Shamima Akter, Anupam Das, Safayet Hossain Piyel, Md. Mahfuj Islam, Sarowar Haque, Anandha Mozumder, Roni Mia, M. Shaminur Rahman, Mohammad Imtiaj Uddin Bhuiyan, Sharmin Akter, M. Nazmul Hoque, Tofazzal Islam, Md. Golzar Hossain

**Affiliations:** 1 Livestock Research Institute 560480, Dhaka, Bangladesh; 2 Department of Microbiology and Hygiene, Bangladesh Agricultural University 54492, Mymensingh, Bangladesh; 3 Department of Microbiology, Jashore University of Science and Technology 421984, Jashore, Bangladesh; 4 Department of Environmental Health Science, University of Georgia 1355, Athens, USA; 5 Department of Physiology, Bangladesh Agricultural University 54492, Mymensingh, Bangladesh; 6 Molecular Biology and Bioinformatics Laboratory, Department of Gynecology, Obstetrics and Reproductive Health, Gazipur Agricultural University 198780, Gazipur, Bangladesh; 7 Institute of Biotechnology and Genetic Engineering, Gazipur Agricultural University 198780, Gazipur, Bangladesh; DOE Joint Genome Institute, Berkeley, California, USA

**Keywords:** fowlpox virus, vaccine strain, genome sequencing, LRI Bangladesh

## Abstract

We report the complete genome sequence of the live attenuated fowlpox virus vaccine strain from the Livestock Research Institute, Bangladesh. This sequence will be valuable for comparative genomic studies with field strains, and for developing improved vaccine seed strategies in the country.

## ANNOUNCEMENT

Fowlpox virus (FPV) (Genus *Avipoxvirus*, Family *Poxviridae*) infection is prevalent worldwide, including in Bangladesh (1). It causes substantial economic losses to the poultry industry through reduced growth rates, decreased egg production, and increased morbidity and mortality (2, 3). Several imported vaccines and a Livestock Research Institute (LRI)-developed live-attenuated vaccine are used in Bangladesh to prevent FPV infection. However, FPV infections are frequently reported in poultry flocks in Bangladesh (1, 4). The LRI vaccine strain has not been sequenced yet. Therefore, we conducted whole-genome sequencing of the LRI-developed FPV vaccine strain (Buddett) to facilitate comparative analyses with circulating field strains.

The FPV vaccine seed strain was obtained on 16 September 2025 directly from LRI and propagated in 10-day-old embryonated chicken eggs (ECEs) via the chorioallantoic membrane (CAM), following a previously described protocol (4). Allantoic fluids from FPV-inoculated ECEs were collected, and viral genomic DNA was extracted using the TIANamp Virus DNA/RNA Kit (Tiangen, China) according to the manufacturer’s instructions. Shotgun metagenomic, moderate-coverage whole-genome sequencing (WGS) libraries were prepared using a modified iNextEra protocol. Key parameters, including input DNA quantity, bead-linked transposase volume, and PCR cycle number, were optimized to improve library yield, complexity, and genome coverage (5, 6). Library tagmentation was performed using Illumina bead-linked transposomes (Illumina DNA Library Prep, Illumina, Inc.), followed by paired-end sequencing (2 × 150 bp) on the Illumina NovaSeq X Plus platform (Novogene, USA).

A total of 34.53 million raw paired-end read quality was assessed using FastQC v0.11.9, and low-quality bases and adapter sequences were removed using Trimmomatic v0.39, retaining reads ≥50 bp in length. Host-derived reads were removed by mapping filtered reads to the *Gallus gallus* reference genome (GCA_000002315.5; GRCg6a) using BBMap (7). Unmapped reads were then aligned to the FPV reference genome (GenBank accession: NC_002188.1) using BWA-MEM v0.7.17 (8). Alignment files were sorted and indexed with SAMtools v1.12 (9), followed by variant calling and processing performed using FreeBayes v1.3.6 (10), and a high-confidence consensus genome was generated using BCFtools v1.12 (11) for variant filtering, normalization, and consensus sequence construction. Genome annotation was performed using Prokka v1.14.6 (12), and terminal genomic regions were defined through comparison with previously published FPV genomes (13). All analyses were conducted using default software parameters.

Host DNA removal was highly efficient, with 95.24% of reads mapping to the chicken reference genome and 4.76% retained for downstream analysis. Alignment of host-filtered reads to the FPV reference genome yielded a complete viral consensus sequence. In total, 159,212 reads aligned to the FPV genome, spanning 286,890 bp and covering 99.42% of the reference genome, with a mean sequencing depth of approximately 61.32×. Uncovered positions (0.58%) were missing regions with no read depth and were filled with reference bases during consensus generation. High sequencing accuracy was indicated by a mean base quality score of 39.3 and a mean mapping quality of 54.2, supporting the reliability of the consensus assembly. Comparative BLAST analysis revealed 99.95% and 99.89% nucleotide sequence identity with the previously reported FPV vaccine strain FWPV-S (GenBank accession: MW142017.1, Australia) and a local FPV strain (GenBank accession: PV982279.1, Bangladesh), respectively. Genome annotation identified 260 open reading frames (ORFs) with a GC content of 30.89% and identical 9.5-kb inverted terminal repeat regions at both the 5′ and 3′ ends, consistent with previous reports (14).

This study reports the complete genome sequence of an FPV vaccine strain from Bangladesh, providing genomic data that may support future vaccine improvement strategies and contribute to improved control of fowlpox outbreaks in poultry populations.

## Data Availability

The complete genome sequence of the FPV vaccine strain (FPV_MGH-AD02) has been deposited in GenBank, BioSample, BioProject, and SRA under accession numbers PX852200, SAMN54559797, PRJNA1401287, and SRR38172611, respectively. The analysis pipeline is available at https://github.com/SShaminur/Viral-Variant-and-Concensus-Calling-Pipeline-from-Metagenome-virvarcall-pipeline.
